# Genome, Environment, Microbiome and Metabolome in Autism (GEMMA) Study Design: Biomarkers Identification for Precision Treatment and Primary Prevention of Autism Spectrum Disorders by an Integrated Multi-Omics Systems Biology Approach

**DOI:** 10.3390/brainsci10100743

**Published:** 2020-10-16

**Authors:** Jacopo Troisi, Reija Autio, Thanos Beopoulos, Carmela Bravaccio, Federica Carraturo, Giulio Corrivetti, Stephen Cunningham, Samantha Devane, Daniele Fallin, Serguei Fetissov, Manuel Gea, Antonio Giorgi, François Iris, Lokesh Joshi, Sarah Kadzielski, Aletta Kraneveld, Himanshu Kumar, Christine Ladd-Acosta, Geraldine Leader, Arlene Mannion, Elise Maximin, Alessandra Mezzelani, Luciano Milanesi, Laurent Naudon, Lucia N. Peralta Marzal, Paula Perez Pardo, Naika Z. Prince, Sylvie Rabot, Guus Roeselers, Christophe Roos, Lea Roussin, Giovanni Scala, Francesco Paolo Tuccinardi, Alessio Fasano

**Affiliations:** 1 Theoreo srl spin off company of the University of Salerno, Via degli Ulivi, 3, 84090 Montecorvino Pugliano (SA), Italy; scala@theoreosrl.com; 2 Faculty of Social Sciences, Health Sciences Unit, Tampere University, Arvo Ylpön Katu 34, 33014 Tampere, Finland; reija.autio@tuni.fi; 3 Bio-Modeling System, 3, Rue De L’arrivee. 75015 Paris, France; thanos.beopoulos@bmsystems.org (T.B.); manuel.gea@bmsystems.org (M.G.); francois.iris@bmsystems.org (F.I.); 4 Department of science medicine translational, University of Naples Federico II, Via Pansini 5, 80131 Naples, Italy; carmela.bravaccio@unina.it; 5 Promete srl, Piazzale Tecchio 45, 80125 Napoli, Italy; federica.carraturo@unina.it (F.C.); tuccinardi@promete.it (F.P.T.); 6 Azienda Sanitaria Locale (ASL) Salerno, Via Nizza, 146, 84125 Salerno (SA), Italy; corrivetti@gmail.com; 7 National University of Ireland Galaway, University Road, Galaway, Ireland; stephen.cunningham@nuigalway.ie (S.C.); lokesh.joshi@nuigalway.ie (L.J.); geraldine.leader@nuigalway.ie (G.L.); arlene.mannion@nuigalway.ie (A.M.); 8 Massachusetts General Hospital, Fruit Street, 55, Boston, MA 02114, USA; SDEVANE@mgh.harvard.edu (S.D.); smkadzielski@mgh.harvard.edu (S.K.); 9 John Hopkins School of Public Health and the Wendy Klag Center for Autism and Developmental Disabilities, 615 N. Wolfe St, Baltimore, MD 21205, USA; dfallin@jhu.edu (D.F.); claddac1@jhu.edu (C.L.-A.); 10 Laboratory of Neuronal and Neuroendocrine Differentiation and Communication, Inserm UMR 1239, Rouen University of Normandy, 25 rue Tesnière, 76130 Mont-Saint-Aignan, France; Serguei.Fetissov@univ-rouen.fr; 11 Medinok Spa, Via Palazziello, 80040 Volla (NA), Italy; a.giorgi@perfexia.it; 12 Division of Pharmacology, Utrecht Institute for Pharmaceutical Sciences, Faculty of Science, Utrecht University, Universiteitsweg 99, 3508 TB Utrecht, The Netherlands; a.d.kraneveld@uu.nl (A.K.); l.n.peraltamarzal@uu.nl (L.N.P.M.); p.perezpardo@uu.nl (P.P.P.); n.z.prince@uu.nl (N.Z.P.); 13 Danone Nutricia Research, Uppsalalaan, 12, 3584 CT Utrecht, The Netherlands; Himanshu.KUMAR@nutricia.com (H.K.); Guus.ROESELERS@danone.com (R.G.); 14 Institut National de Recherche Pour L’agriculture, L’alimentation et L’environnement (INRAE), AgroParisTech, Micalis Institute, Université Paris-Saclay, 78350 Jouy-en-Josas, France; Elise.Maximin@inrae.fr (E.M.); laurent.naudon@inrae.fr (L.N.); sylvie.rabot@inrae.fr (S.R.); lea.roussin@inrae.fr (L.R.); 15 Consiglio Nazionale delle Ricerche (CNR), Piazzale Aldo Moro, 7, 00185 Roma, Italy; alessandra.mezzelani@itb.cnr.it (A.M.); luciano.milanesi@itb.cnr.it (L.M.); 16 Euformatics, Tekniikantie, 02150 Espoo, Finland; christophe.roos@euformatics.com; 17 European Biomedical Research Institute of Salerno (EBRIS), Via S. de Renzi, 3, 84125 Salerno (SA), Italy; afasano@mgh.harvard.edu

**Keywords:** microbiome, metabolomics, autism, study design, biomarker discovery, precise medicine

## Abstract

Autism Spectrum Disorder (ASD) affects approximately 1 child in 54, with a 35-fold increase since 1960. Selected studies suggest that part of the recent increase in prevalence is likely attributable to an improved awareness and recognition, and changes in clinical practice or service availability. However, this is not sufficient to explain this epidemiological phenomenon. Research points to a possible link between ASD and intestinal microbiota because many children with ASD display gastro-intestinal problems. Current large-scale datasets of ASD are limited in their ability to provide mechanistic insight into ASD because they are predominantly cross-sectional studies that do not allow evaluation of perspective associations between early life microbiota composition/function and later ASD diagnoses. Here we describe GEMMA (Genome, Environment, Microbiome and Metabolome in Autism), a prospective study supported by the European Commission, that follows at-risk infants from birth to identify potential biomarker predictors of ASD development followed by validation on large multi-omics datasets. The project includes clinical (observational and interventional trials) and pre-clinical studies in humanized murine models (fecal transfer from ASD probands) and in vitro colon models. This will support the progress of a microbiome-wide association study (of human participants) to identify prognostic microbiome signatures and metabolic pathways underlying mechanisms for ASD progression and severity and potential treatment response.

## 1. Introduction

Autism Spectrum Disorder (ASD) is a lifelong neurodevelopmental disorder characterized by deficits in social communication and social interaction, in addition to restricted, repetitive patterns of behavior, interests, or activities [1]. As a spectrum disorder, its symptoms may range from mild to severe. Some children may have strong language and intellectual abilities whereas others may not be verbal and may require lifelong care. Globally, ASD incidence has shown a 35-fold increase compared to the 1960s and 1970s, when the first epidemiological studies were conducted [2,3,4,5,6]. As documented by the Centers for Disease Control and Prevention, ASD affects 1 in 54 children in USA [7] and 1 in 89 in Europe [8].

Children born in a family with an affected sibling show a ten-fold higher risk of developing the condition [9]. These data, including a large-scale exome sequencing study [10], suggest a combination of non-Mendelian genetic and environmental factors in ASD pathogenesis [11,12]. One environmental factor that is emerging as important for ASD risk is the immune system [13,14,15,16]. Indeed, individuals with ASD show increased expression of genes encoding mediators of the innate immune response [15]. Low-grade systemic inflammatory events [17,18], combined with the hypofunction of protective, anti-inflammatory mechanisms [19], lead to mechanisms related to the biochemical and neuroanatomical characteristics associated with autism pathogenesis [20,21,22]. Immunoregulation, in addition to dysregulated immunoregulation, particularly during the first 1000 days of life [23], are guided by the gut microbiome. Low-grade systemic inflammatory responses can lead to psychiatric disorders, such as ASD and psychological stress, which leads to further inflammation through pathways involved in the intestinal microbiota homeostasis [24,25].

Many individuals with ASD have symptoms of associated comorbidities. These include medical comorbidities, such as gastrointestinal (GI) and immune system disorders (namely gut dysbiosis, susceptibility to infections, and increased prevalence of autoimmune disorders), sleep problems, feeding problems and epilepsy; comorbid psychopathology, including Attention-Deficit/Hyperactivity Disorder (AD/HD), anxiety, mood problems, disruptive behavior; and developmental comorbidity, namely the presence of an intellectual disability.

A large number of individuals are living with ASD. Thus, ASD is a serious public health concern. The medical expenditure for ASD is higher than that for cancer, heart disease, and stroke combined [26,27]. Blumberg et al. [28] suggested that the recent increase in prevalence could be in part attributed to greater recognition and awareness, or changes in clinical practice or service availability. However, these changes are not sufficient to explain this phenomenon [6]. ASD is a multifactorial disorder; therefore, the contribution of environmental factors could explain its development in addition to why different therapeutic approaches, based on gene/environment interaction theory, have achieved conflicting results [29,30,31]. The GEMMA (Genome, Environment, Microbiome and Metabolome in Autism) team hypothesizes that many environmental factors can alter intestinal microbiota composition and activity, causing epigenetic modifications, changes in the metabolome profile, and increases in intestinal barrier permeability and macromolecule trafficking with the alteration of immune responses, thus contributing to the progression and development of ASD. Furthermore, it is hypothesized that the genome/metagenome interaction determines the switch from immune tolerance to immune response. Environmental stimuli, including dietary and microbial factors, can trigger an immune response, such as the neuroinflammation responsible for the behavioral changes that characterize ASD and intestinal inflammation causing its GI comorbidity.

The existence of a gut–brain axis was hypothesized by Bolte in the late 1990s [32]. This was one of the first reports of unhealthy changes in the intestinal tract’s resident community (microbiome), driving both behavioral and GI problems in babies with ASD. Finegold et al. [33] showed that the increase in toxin-producing gut bacteria populations directly affects the brain via the vagus nerve. Currently, changes in gut microbiota of ASD patients with and without GI symptoms are well established [34,35,36], suggesting the potential beneficial effect of fecal transplantation [37,38].

Unfortunately, current large-scale studies of ASD are mainly of cross-sectional study design, which can provide information about associations between microbiome changes and ASD diagnoses but lack the information regarding the temporality of these changes. This limits inferences about causality or utility as an early life biomarker. Indeed, currently, there are no established biomarkers for ASD to enable its clinical diagnosis, which relies on behavioral evaluations. In addition, no medications are currently available to treat the core symptoms of ASD and results from intervention research are mixed [39].

## 2. Study Design

**Figure 1:**
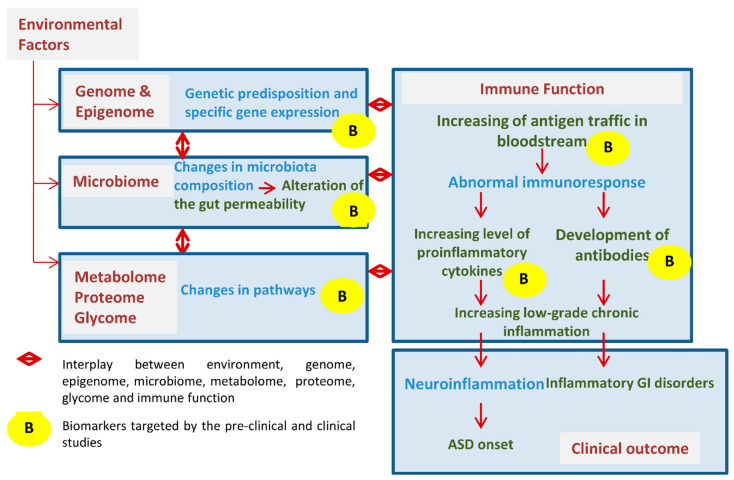
GEMMA (Genome, Environment, Microbiome and Metabolome in Autism) study design.

GEMMA is a multi-center, prospective, open-label, uncontrolled study with an observational and an interventional arm, comprised of collaborators in the European Union and the United States. It is coordinated by the European Biomedical Research Institute of Salerno (EBRIS), in Campania, Italy. GEMMA aims to study genomic, environmental, microbiome, and metabolomic factors that, via the immune system, may contribute to the development of ASD longitudinally. This study offers the potential to identify the mechanisms and/or biomarkers involved in either development or exacerbation of ASD signs and symptoms, which will potentially broaden the range of available therapeutic interventions (Figure 1).

The project includes clinical (observational and interventional trials) and pre-clinical studies. Several pre-clinical studies in humanized murine models (fecal transfer from ASD probands) and in vitro colon models will be performed simultaneously with clinical studies from the moment of recruitment of at-risk infants and their affected ASD sibling. This will support the progress of a microbiome-wide association study (of human participants) to identify prognostic microbiome signatures and metabolic pathways underlying mechanisms for ASD progression and severity and potential treatment response. The preclinical work will provide valuable information regarding validation of specific biomarkers mechanistically linked to the onset of ASD, which will be used to rationalize future patient stratification for primary intervention.

### 2.1. Pre-Clinical Studies

**Figure 2:**
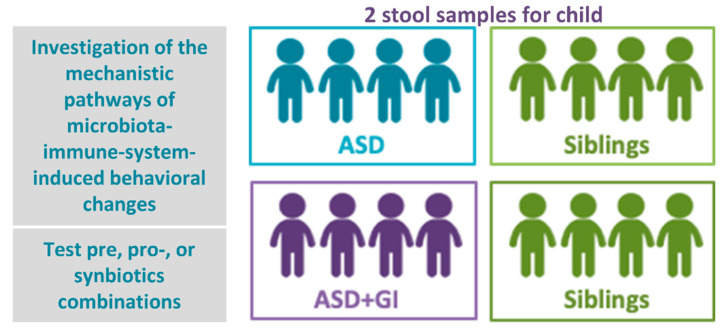
Preclinical studies—development of humanized mouse models by transplanting fecal samples from a well-characterized ASD diagnosed proband (and sibling controls). Four groups of children, each with 4 children, recruited in one center in Europe (Italy): autistic with no GI problems, autistic with GI problems, controls without or with GI problems. Ongoing.

An in vitro micro-scale colon microbiota model or “colon microcosm” that mimics the microbiota of the human colon will be used to investigate effects of various combinations of prebiotic fiber types and probiotics on microbiota composition and functionality. The colon microcosms will be inoculated with fecal samples collected from children diagnosed with ASD with and without a history of GI problems, and from healthy siblings (controls) between the ages of 3 and 14. Samples will be excluded if the participants have used psychotropic medications in the previous 6 months and antibiotics and/or probiotics in the previous 2 months (Figure 2).

The impact of pre- and probiotic combinations on microbiota composition and function will be measured by 16S rRNA gene sequencing, metagenomics, and targeted and untargeted metabolites analyses.

The preclinical studies in murine models will be carried out simultaneously with the other studies for two reasons: to provide further information on validation of specific biomarkers mechanistically linked to the onset of ASD and, consequently, to treat future patients primarily with a more precise intervention.

The preclinical studies in murine models include the development of humanized mouse models through transplanting fecal samples from a well-characterized ASD diagnosed proband (and sibling controls) in the families included in the clinical observational study. The fecal samples will be the same as those collected for the in vitro studies described above. In addition to wild type naïve mice, fecal transplantation will be conducted in genetically vulnerable (PCDH-KO), food allergic (CMA), and valproate-treated (VPA) male mice which have been previously demonstrated to show ASD-like behavior [40,41,42,43]. The natural gut microbiota of these mice will be depleted before transplantation with the human fecal samples. This will be achieved at the age of 3 weeks, with antibiotic treatment followed by a bowel cleansing according to procedures described by Le Roy and coworkers [44]. Fecal transplantation will also be conducted in germ-free male mice, which have abnormal development of the nervous system and show deficits in cognition, social behavior, and stress-related behaviors [45,46,47,48,49,50,51]. The microbial content of human donors will be orally administered to recipient microbiota-depleted or germ-free mice. Behavior, microbiota composition, gut permeability, metabolites, mucosal and systemic immune profiles (intestinal tract, mesenteric lymph nodes, blood, and spleen), neurotransmitters, and neuroinflammation in the brain will be evaluated to investigate the mechanistic evidence of the intestinal microbiota–brain axis in ASD. In addition, ASD-humanized VPA and CMA male mice will be suitable splenocyte donors for transferring their behavioral phenotype to naïve mice to reveal whether the ASD-like phenotype is microbiome-immune system-dependent. A second series of preclinical studies will establish whether correction of intestinal microbial dysbiosis by pre/pro/synbiotic intervention prevents and/or treats ASD-like behavior in ASD murine models. Selected ASD humanized murine models will be fed diets supplemented with selected pre/pro/synbiotics, which will be prescreened using microcosms systems as described above. Those that affect neurodevelopment, behavior, and the GI tract positively will be used in the clinical intervention trial.

### 2.2. Clinical Study

#### 2.2.1. Participants

GEMMA aims to enroll 600 infants younger than 6 months/26 weeks of age, who have an older sibling with ASD for the observational trial. The recruitment will be carried out in three different centers: Irish Center for Autism and Neurodevelopmental Research (ICAN) at the National University of Ireland, Galway, Azienda Sanitaria Locale Salerno (ASL), and satellite centers in Italy, and Mass General Hospital for Children (MGHFC) Lurie Center for Autism in USA. The first study samples must be collected prior to the introduction of solid foods. Children who have started any other therapeutic intervention for ASD will also be excluded from the study. According to our previous experience with similar projects involving two cohorts of infants at-risk of celiac disease [52,53], enrollment will be performed by inviting parents’ of the autistic patients who are followed by the recruitment clinics to participate in the study in the case of newborns, and advertising the study among ASD support and ASD patients’ groups. Moreover, a traditional and social media diffusion plan was created to increase the project knowledge and support. No compensation will be provided to the participant.

Infants aged 18–36 months who were recruited, completed the observational study, developed clinical signs and symptoms of ASD, and whose parents permit the intervention of solely dietary and oral therapy in this study, will be invited to enroll for a follow-on dietary interventional trial.

#### 2.2.2. Observational Trial

This study phase will evaluate infants at high risk of ASD. To achieve the sample size needed for clinical and biological comparisons, and ensure there is a reasonable representation across study sites, each site will target recruitment of 200 infants at high risk of developing ASD, over a 3 year period. All recruited children will undergo regular clinical and laboratory evaluations for 36 months, or more in the case of ASD development based on the annual intermediate clinical data check. Blood, urine, stools, and saliva samples will be collected every 6 months until 36 months of age.

Regarding GI symptoms, children will be evaluated for chronic irregular bowel movements (constipation, diarrhea), encopresis, recurrent abdominal pain, gastroesophageal reflux, and vomiting or food aversion. On these bases, the evaluable target study population (see Table 1 for inclusion/exclusion criteria) will be divided into four groups (depending on their clinical outcome: neuro competent at-risk infants with (NC-GI) and without GI symptoms (NC), ASD infants with GI symptoms (ASD-GI) and ASD infants without GI symptoms (ASD)). Maternal and paternal genomic samples will be taken from parents of infants at the time of enrollment and analyzed to examine inherited and de novo genetic traits.

**Figure 3:**
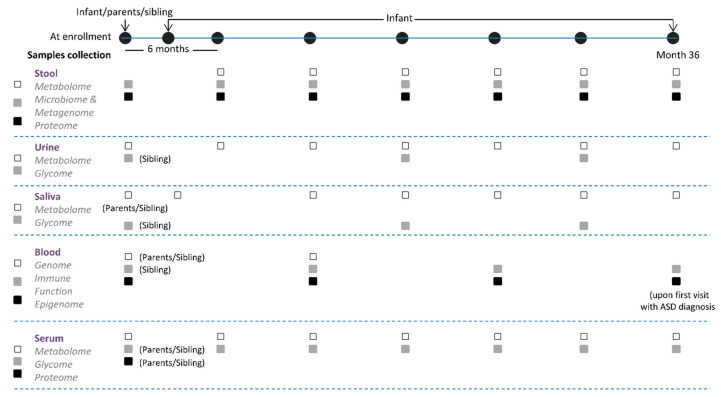
Clinical trials—prospective multi-center observational study evaluating infants at risk of ASD. 600 infants—newborns, less than 6 months of age—at risk, i.e., first-degree relatives of ASD individuals, recruited in US, Italy, and Ireland (200 infants at each site, over a 3-year period). Ongoing.

Autism Diagnostic Observation Schedule (ADOS Toddler) evaluation [54] will be conducted every 6 months by a trained physician, starting at 12 months of age. For children younger than 24 months who test positive in the ADOS Toddler evaluation, the evaluation will be repeated one month later to confirm diagnosis to enroll the infant in an interventional study, and will be used to divide the participants into two groups: ASD children or non-ASD children (typically developing). Typically developing children will be followed until 36 months (see Figure 3) of age and their data will be selected at the end of the study to provide the information for the matched control comparisons.

Additional behavioral assessments include Early Screening for Autism and Communication Disorders and Repetitive Behavior Scale [55,56], Mullen Scales of Early Learning (MSEL) [57], and Vineland Adaptive Behavior Scales (VABS) [58]. These assessments will take place at 24 and 36 months of age.

#### 2.2.3. Interventional Trial

**Figure 4:**
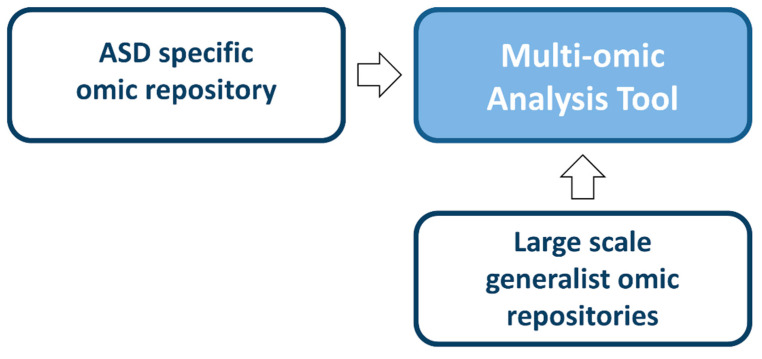
Interventional trial—prospective, multi-center, open-label, uncontrolled study to evaluate the effects of pre/pro/synbiotics supplementation in infants with a diagnosis of ASD. Forty children between the ages of 18 and 36 months diagnosed with ASD from the observational trial will be recruited (with parental consent).

This prospective, multi-center, open-label, uncontrolled study (Figure 4) evaluates the effects of pre/pro/synbiotics supplementation started in infants between the ages of 18 and 36 months with a diagnosis of ASD.

A minimum of 40 children diagnosed with ASD from the observational trial will be recruited to the interventional trial, subject to parental consent. Each ASD infant will undergo a Positron Emission Tomography (PET) scan of their brain, at the beginning and end of the interventional study, to obtain information on the degree of neuro-inflammation and the potential effect of the intervention. The presence/absence of GI symptoms will also be evaluated.

Clinical and laboratory assessments will be made at the time of enrollment, monthly for 3 months, and then at the conclusion of the 6 month study.

The study was approved by the relevant ethics committee of each enrolling country. In particular: CE Campania Sud (IRB n.30/2019) for Italy; Partners Human Research (IRB ver.01/04/2019) for USA; and Clinical Research Ethics Committee of Galway University Hospital (IRB n. C.A. 2127/19) for Ireland. A written consent form will be signed by each participant or their legal representative. The clinical trial was registered with the access number: NCT04271774 (https://clinicaltrials.gov).

### 2.3. Data Collection

The timetables of data and sample collection are shown in Supplementary Tables S1–S3 and in Figure 3. Pre-screening may occur verbally via a telephone call or in person at a hospital clinic. Parents who wish to enroll their infant in the study will provide proof of ASD diagnosis for the child with the condition. Each clinical site will be responsible for its own data collection and management. Data will be monitored remotely for completeness and coherence by the project management team. All data will be collected through a secure, internet-based electronic capture system (REDCap) [59]. Participating institutions will be granted access to the electronic storage module with all appropriate forms and schedules pre-loaded. Each institution will only be able to view data for participants enrolled at their site. The coordinating center will be granted access to unidentified data from all sites through the electronic data capture system.

#### Parental and Child Questionnaires

An online questionnaire will be filled out by the family every month during the 6 months of intervention. Moreover, it will be filled out by the family at 9, 15, 21, 27, and 33 months of age and will include infant dietary data and the use of concomitant medication (vaccinations and antibiotics), demographics, infant clinical history including but not limited to infections, onset of food intolerance, and chronic stress. Parents will also be requested to complete the Aberrant Behavior Checklist (ABC) at all visits and at 9 months. Assessment scales for infant development (Rome 3 Criteria GSRS, ADOS-Toddler, Mullen scale, CGI-S, CGI-I) will be assessed, during the interventional trial, every month for the first three months of the study, and three months later at the final 6-month study visit.

### 2.4. Serological Markers

#### 2.4.1. Whole Blood

Blood sampling will take place at enrollment, 0–6 months post birth, and at 12, 18, 24, 30, and 36 months of age. Blood for hematology, biochemistry evaluation, biomarker identification, and genetic assays will be collected from infants fasting for at least 3 h. The blood collection procedure is as follows: via umbilical cord for infants enrolled during gestation, or by heel sampling at enrollment (at 0–6 months of at-risk infant age), 1 mL every 6 months from 12 months of age, and finally at development of ASD or at the 36-month (observational study termination visit). The assays that will be performed on blood include whole genome sequencing on infants that develop ASD. Furthermore, DNA methylation (DNAm) will be measured among infants that develop ASD using samples obtained at three time points: (1) enrollment, (2) the time of ASD diagnosis, and (3) the completion of the interventional trial. In preparation for future testing, a biorepository will be established. A blood sample will be taken after 12 months for peripheral blood mononuclear cell (PBMC) isolation and immune profiling analysis.

Moreover, serum testing will take place at enrollment of the clinical study, 0–6 months post birth, and at 12, 18, 24, 30, and 36 months of age. A quantity of 500 uL of serum will be taken from the infant fasting for 3 h to evaluate serum zonulin (20 μL); serum IgA and IgG anti-gliadin and anti-casein antibody determination (40 μL) by the ELISA method; serum pro-inflammatory cytokines, including cIL-1, TNF-α, IFN-, IL-10, IL-12, IL-6, IL15, and IL-8 by Bioplex assay (75 μL) [60]; and cytokine gene expression by RT-PCR.

If ASD is developed, whole genome sequencing of the infant and of both parents will be performed.

#### 2.4.2. Stool, Urine, and Saliva Samples

Every 3 months after enrollment, urine and stool samples will be collected at home and shipped to the laboratory for biomarker assay. Because patient samples are divided according to the presence and absence of a history of GI problems with an onset earlier than the initiation of toilet training, the aim is to select children with a biological rather than psychological etiology of GI discomfort. The control group includes fecal samples from age- and gender-matched control participants without previous diagnosis of a neurodevelopmental disorder and without a history of GI problems. Exclusion criteria for the use of patients and control samples include the use of psychotropic medication for the previous 6 months and antibiotics and/or probiotics for the previous 2 months.

Saliva samples will be taken for microbiome and glycome (the whole set of carbohydrates) analysis. Moreover, when possible subsequent analysis/correlation with blood biomarker levels either within this protocol or at a later timepoint will be performed. The saliva samples will be analyzed for pro-inflammatory markers and for six inflammatory markers selected from a previous study [61].

## 3. Factor of Interest

### 3.1. Environmental

Birthing delivery mode, method of infant feeding, and introduction of solid food and allergen to the infant diet could represent potential environmental factors causing ASD, so these will be recorded for each enrolled participant [53,62,63]. A diary of antibiotic usage will be completed by the parents monthly for the first year of life, and food diaries completed at the same time points will assess duration of breastfeeding (or other preferred feeding mode).

After the first year of life, detailed, although less frequent, records of antibiotic use will be obtained with each stool sample for the remaining duration of the study. Although the food diary will be discontinued at this time, pertinent dietary habits of both the mother and child will continue to be carefully recorded approximately every six months.

Moreover, details such as antibiotic exposure in early infancy, family living address, exposure to pets, and gastrointestinal sporadic symptoms will also be recorded.

### 3.2. Genetics

Currently, ASD heritability based on mono- and dizygotic twins is estimated to be about 79–80% [64,65]. Concordance is higher in monozygotic twins than dizygotic twins.

First-degree relatives of ASD probands show behavioral or cognitive features associated with autism intensification, such as language and social dysfunction, albeit in lesser forms [66], known as “broader phenotype” [67,68,69,70].

ASD-related social impairments are heritable [71] and increase in unaffected parents and children of autistic probands [72]. Several studies evaluating sub-threshold ASD traits in population cohorts suggested that different components, separately representing language, social function, and repetitive or stereotyped behaviors contribute to ASD [73,74,75]. Globally, these results suggest that ASD features represent a continuum of function that may be inherited in distinct patterns. This is consistent with the knowledge that specific genetic factors contribute to the development and function of specific brain structures, and that distinct brain circuits may underlie different components of autism [76].

Moreover, several genetic syndromes, including Fragile X, Tuberous Sclerosis, Joubert Syndrome and Smith Lemli Opitz, are known to cause, although with low penetrance, ASD-like symptoms [76,77]. This evidence further favors a genetic cause for ASD [78].

To assess the contribution of genetic risk factors, in combination with exposure and other omics data, we will perform whole genome sequencing in all participants with ASD, in addition to their parents. This will enable investigation of both common and rare variants that contribute to ASD risk.

### 3.3. GI Microbiome and Metabolome

The complete understanding of the host-GI microbiome genomics is necessary for the prediction of the activation of metabolic pathways linked with functional and clinical outcomes. Urine and stool samples will be collected and analyzed for a metabolomic phenotype of the GI microbiota and metabolomics profiles, including metabolites linked to bacterial activity in ASD. The metabolomic profiles, exploratory semi-quantitative assays, and targeted quantitative assays will be conducted throughout the study. Systems models of integrated metabolomic phenotypes will be created with the statistically linked data between the microbiota and immune function. By conjoining microbiota data and metabolomic data we will be able to understand the functions associated with GI comorbidity of children with ASD and the ASD patient-derived microbiota. Dietary interventions with pre/pro/synbiotics will be created based on the results found in the humanized mouse models undergoing fecal transplantation with ASD-specific microbiota.

### 3.4. Immune Function

The peripheral blood mononuclear cells (PBMCs) will be harvested and studied for immune functions. Standard flow cytometry will be used to measure monocyte and T-cell intracellular cytokine patterns. By looking at the T cell populations in children with ASD with and without GI symptoms we will be able to understand what cellular activation and functional alterations are present. Assays performed on serum samples collected at clinical visits include: serum zonulin determination; serum IgA and IgG anti-gliadin and anti-casein determination by the ELISA method; serum pro-inflammatory cytokines, including IL-1, TNF, IFN, IL-10, IL-12, IL-6, IL-15, and IL-8 by Bioplex assay; and cytokine gene expression by RT-PCR. Protein glycosylation is directly involved in nearly every biological process presenting altered glycosylation as potential biomarkers in many human conditions including inflammation, diabetes, rheumatism, cancer, and neurological disorders [79]. Serum immunoglobulin glyco-signaturing will be performed using lectin microarrays and anti-glycan ELISA profiling. Saliva samples will also be analyzed for proinflammatory cytokines and inflammatory markers selected from a previous study [61], and altered glycan and glycation products.

## 4. Statistical Approach

### 4.1. Statistical Methods

Categorical data will be described using frequency counts and percentages, and continuous data with mean, standard deviation, median, minimum, and maximum. Both parametric and non-parametric methods will be used to analyze the data. All omics data will be preprocessed with appropriate methods. The multi-omics analyses will be performed with statistical tests, regression models, and more advanced methods such as sparse canonical correlation analysis and random forest analysis. The false discovery rate (FDR) method will be used to adjust the *p*-values for multiple comparisons. Statistical significance will be set at *p* < 0.05.

### 4.2. Power Analysis

We estimated a conservative drop-off of 20% based on our previous experience with similar projects involving two cohorts of infants at-risk of celiac disease, one that has been followed for 10 years with a 20% drop-off [52] and a second that has been followed up to 7 years with a 12% drop-off [53]. The final cohort will comprise approximately 500 at-risk infants. Expected prevalence of ASD at 18–24 months in first-degree relatives is 14.7% [9], so in this study we estimate 73 ASD diagnoses. The expected prevalence of GI symptoms in an ASD population is 60% [80], so we anticipate 44 of the 73 ASD diagnosed infants to also present GI symptoms.

### 4.3. Developing an Integrative Multilevel Model to Predict ASD

Path a: A causal path between the genetics and the final effect on the onset of ASD, with the microbiome as the mediator (a1 × a2), so that the intestinal microbiome will be altered for the genetics to ultimately influence the development of the disorder (microbiome-mediated epigenetic pressure).

Path b: A causal path of the microbiome’s effect on ASD mediated (b1 × b2) by the metabolic activity of intestinal bacteria, which, through the production of metabolites, affects the intestinal transcriptome and proteome. Including a bidirectional path between microbiome and metabolomics to capture how metabolism could alter the microbial metabolism and ultimately the risk for ASD.

Path c: A bidirectional path between the transcriptome and the microbiome because their causal relationship will depend on each other.

Path d: A direct relationship between lifestyle factors and the onset of the disorder mediated (d1 × d2) by alterations in the microbiome.

Path e: The environment affecting the onset of the disorder as a mediator and affecting the composition of the microbiome and resulting metabolome, in turn, influencing gene and protein expression.

Full Model: Time points will be clustered within individual characteristics; analyzing all of the paths listed above, individuals will be clustered within families, and families within regions.

## 5. Discussions and Conclusions

The implementation of primary prevention strategies for ASD via manipulation of the intestinal microbiota represents a complete paradigm shift of treatment and prevention of ASD. The identification of specific ASD metagenomics and metabolomics phenotypes can also help to define additional biomarker-based diagnostic tools and therapeutic interventions. Additionally, GEMMA’s biorepository will encourage future epigenetic studies and validation of biomarkers. Our findings may also impact the prevention and treatment of other neuroinflammatory conditions.

## Figures and Tables

**Table 1:** Inclusion and exclusion criteria for the observational trial.

Inclusion Criteria (All Must be Met)	Exclusion Criteria (Participant Will be Excluded from the Study if Any of the Criteria are Met)
Healthy newborns or infants First-degree relatives of ASD participant (at least one sibling affected by ASD) Younger than 6 months/26 weeks Have never received solid food (elementary formula feeding permitted)	Newborns with significant health issues that require surgical treatments or continuous medical treatments and/or surgical treatments Infants older than 6 months/26 weeks Infants who have been introduced to solid food (including occasional use) Severe GI problems requiring immediate treatment (life-threatening) Severely underweight/malnourished children Dietary restriction in the previous 3 months Tube feeding Drugs since birth which may affect the biomarkers being assessed, for example Antibiotics within one month prior to enrollment; antibiotics used as a continuous course for ≥28 days prior to enrolment

## Supplementary Materials

The following are available online at https://www.mdpi.com/2076-3425/10/10/743/s1, Table S1: Information that will be collected from the enrolled participant during the project. Table S2: Samples that will be collected from the enrolled participants divided for project time. Table S3: Psychiatric evaluation of the enrolled participants.
