# Supplementary material for: Genome, Environment, Microbiome and Metabolome in Autism (GEMMA) Study Design: Biomarkers Identification for Precision Treatment and Primary Prevention of Autism Spectrum Disorders by an Integrated Multi-Omics Systems Biology Approach
Source: Brain Sci. 2020 Oct 16;10(10):743. doi: 10.3390/brainsci10100743 (PMC7603049; doi:10.3390/brainsci10100743)
Supplement: Supplementary file 1 [file brainsci-10-00743-s001.pdf]

Table S1. Information that will be collected from the enrolled participant during the project.

Months	Enrollment Visit 0-<6 MTHS	Family Contact 9 MTHS	Clinic Visit 12 MTHS	Family Contact 15 MTHS	Clinic Visit 18 MTHS	Family Contact 21 MTHS	Clinic Visit 24 MTHS	Family Contact 27 MTHS	Clinic Visit 30 MTHS	Family Contact 33 MTHS	End of Study Visit 36 MTHS
Informed Consent (from parents)	X										
Demographics (mother and child)	X										
Parent medical history and parent blood sampling:<br>● maternal risk factors for and history of autoimmune and psychological disorders/infections<br>● medications/drugs during pregnancy<br>● adverse experiences during gestation<br>● samples including genomic samples	X										
Peri-natal information<br>● mode of baby delivery	X										

Table S2. Samples that will be collected from the enrolled participants divided for project time.

Months	Enrollment Visit 0-<6 MTHS	Family Contact 9 MTHS	Clinic Visit 12 MTHS	Family Contact 15 MTHS	Clinic Visit 18 MTHS	Family Contact 21 MTHS	Clinic Visit 24 MTHS	Family Contact 27 MTHS	Clinic Visit 30 MTHS	Family Contact 33 MTHS	End of Study Visit 36 MTHS
Vital Signs (blood pressure, pulse, respiration rate, temperature)	X		X		X		X		X		X
Blood sample (via umbilical cord or heel sampling): Hematology, biochemistry, autoantibodies, genomic, biomarkers	X										
Blood sample: biomarkers			X		X		X		X		
Blood Sample (hell sampling): Hematology, biochemistry, genomics, biomarkers											X
Saliva Samples			X	X	X	X	X	X	X	X	X
Urine Samples	X		X	X	X	X	X	X	X		X
Stool Samples	X	X	X	X	X	X	X	X	X	X	X
Record Medical Events	X		X	X	X	X	X	X	X	X	X
End of Follow up Data Collection			(X)	(X)	(X)	(X)	(X)	(X)	(X)	(X)	(X)

Table S3. Psychiatric evaluation of the enrolled participants.

Months	Enrollment Visit 0-<6 MTHS	Family Contact 9 MTHS	Clinic Visit 12 MTHS	Family Contact 15 MTHS	Clinic Visit 18 MTHS	Family Contact 21 MTHS	Clinic Visit 24 MTHS	Family Contact 27 MTHS	Clinic Visit 30 MTHS	Family Contact 33 MTHS	End of Study Visit 36 MTHS
• Apgar score<br>• possible peri-natal distress<br>• prematurity											
Infant medical history/status including:<br>• infections<br>• onset of food intolerance<br>• chronic stress etc.	X	X	X	X	X	X	X	X	X	X	X
Online Questionnaire to be completed by parents (monthly up to 12 months age) then 3 months thereafter	X	X	X	X	X	X	X	X	X	X	X
Aberrant Behavior Checklist		X	X	X	X	X	X	X	X	X	X
ADOS-Toddler			X		X		X		X		X
MSEL, VABS							X				X
CGI-5			X		X		X		X		X
CG-I			X		X		X		X		X
Infant concomitant medication review including vaccinations and antibiotics (online questionnaire 3 months)	X		X	X	X	X	X	X	X	X	X
Infant dietary Review (online questionnaire at 3 months)	X		X	X	X	X	X	X	X	X	X
Physical Examination including height, weight	X		X	X	X	X	X				X
